# Editorial: Personalized medicine for neuromuscular disorders

**DOI:** 10.3389/fcell.2023.1329048

**Published:** 2023-11-16

**Authors:** Marc Bartoli, Rachel M. Bailey, Kathrin Meyer, Florian Barthélémy

**Affiliations:** ^1^ Institut national de la santé et de la recherche médicale, Marseille Medical Genetics, U1251, Aix Marseille University, Marseille, France; ^2^ Division of Child Neurology, Department of Pediatrics, University of Texas Southwestern, Dallas, TX, United States; ^3^ Center for Alzheimer’s and Neurodegenerative Diseases, University of Texas Southwestern, Dallas, TX, United States; ^4^ Center for Gene Therapy, Abigail Wexner Research Institute at Nationwide Children’s Hospital, Columbus, OH, United States; ^5^ Department of Pediatrics, The Ohio State University, Columbus, OH, United States; ^6^ Center for Duchenne Muscular Dystrophy at UCLA, Los Angeles, CA, United States; ^7^ Department of Microbiology, Immunology and Molecular Genetics, David Geffen School of Medicine, College of Letters and Sciences, University of California, Los Angeles, Los Angeles, CA, United States

**Keywords:** muscle, motor neuron, gene editing, gene transfer, drug repurposing, neuromuscular disease (NMD), disease modeling, therapy screening

Conducting research on rare neuromuscular disorders is challenging due to the heterogeneity of diseases presentations from multi-systemic organ presentation to impacting specific groups of cells or tissues. Furthermore, most of these diseases face several limitations: large heterogeneity of patient presentation, incomplete penetrance, lack of natural histories or scarcity of patients. Indeed, neuromuscular disorders constitute a diverse group of diseases that impact nerves and/or muscles, resulting in various clinical manifestations including, but not limited to, delayed motor function, muscle weakness and atrophy, and movement impairments. The field has beneficiated from all the recent and significant advancements in genomics (with the continuous advancement of sequencing technologies), in stem cell biology (with the creation of new cellular models through the generation of induced pluripotent stem cells) and in molecular biology (with the emergence of gene and RNA editing technologies). Specifically, most of these diseases have the ability to access or obtain multi-omics data, thanks to high throughput sequencing technologies, enabling a comprehensive analysis of these diseases like never before and paving the way for the emergence of innovative and targeted therapeutic approaches. Undeniably, the marketing of orphan drugs (drugs specifically designed for a rare condition) suffer greatly from insufficient research and funding to provide the understanding and knowledge to adequately address these unmet medical needs. Thus, pharmacotherapies typically remain the treatment of choice but are rarely specific and can elicit numerous adverse effects. However, with the advent of promising technologies such as gene transfer or gene and RNA editing, personalized and precise medicine appears to be attainable for a substantial subset of these disorders. In this special emphasis Research Topic, we aimed to highlight advances in different aspects of “*personalized medicine for neuromuscular disorders*” by collecting 6 original articles, 1 brief research report and a review, all focusing on different diseases but exploring the challenges and hopes of this highly dynamic field of research from drug discovery to clinical trials.

One of the most important considerations when working with rare diseases is to understand the etiology, presentation, and natural history of the disease. Often, mutations in one gene can cause multiple diseases with different presentation or even mode of actions. We included a study by Cesar ekt al., that presents an extensive analysis of the cardiac involvement in a group of ultrarare diseases called laminopathies caused by mutations in *LMNA*. They showed unequivocally that arrhythmias and sudden cardiac death (SCD) occur frequently in LMNA-related muscular dystrophy. Using longitudinal data obtained in several families with different disease presentations, they showed the vital importance of a personalized treatment plan that would benefit from implementing cardiac monitoring to mitigate the risks. This should pave the way to genetic counseling resources and clinical guidelines that are needed to standardize treatment and mitigate the risk of SCD.

Genetic manipulation and cellular reprogramming have revolutionized the field by addressing the primary obstacle encountered in rare disease research, which is the need for relevant and patient-derived models. In this context, we recommend the works conducted by Rojas et al., Rossiaud et al., and Almeida et al., which accentuate the power of tissue-relevant cellular models exhibiting a particular mutation to precisely simulate specific disease characteristics and facilitate the establishment of targeted individualized medicine.

Rojas et al. study used reprogramming technologies to generate mature astrocytes from control and a patient line carrying an A90V mutation in *TARDBP* (encoding the protein TDP43). This mutation has been linked to amyotrophic lateral sclerosis (ALS) and frontotemporal dementia (FTD). The authors were able to show that patients’ mature astrocytes display abnormal TDP43 localization, nuclear depletion, and cytoplasmic accumulation as well as mild inflammatory manifestations. The study investigated preventing motor neuron death by neutralizing the inorganic polyphosphate, released by patients’ astrocytes, with the PAMAM dendrimer G4-PAMAM-NH2.

The study by Rossiaud et al., analyzed the phenotype of myotubes derived from the Glycogen Storage Disease Type III (GSDIII) CRISPR hiPSC line, focusing on the expression and activity of the glycogen Debranching Enzyme (GDE) and glycogen content of GSDIII CRISPR and control myotubes. Results showed the absence of GDE expression on mutated skeletal myotubes and a significant decrease in glucose generated by mutated myotubes.

Almeida et al., created four antisense sequences targeting the DMPK mRNA 3′UTR region to hinder CUG repeats found in Myotonic Dystrophic type 1 patient cell models. These sequences were added to the mU7 gene and vectorized in AAV8. One week after treatment, the number of RNA foci per nucleus was decreased, affecting MBNL1 protein localization. In addition, two of the gene therapy constructs (3′CTG and 20CTG) significantly impacted the transcriptome of treated myotubes, identifying differentially expressed genes related to immune response, and partially correcting abnormal alternative splicing in DM1 myotubes.

Despite their limited representation of disease severity and trajectory in comparison to human, the neuromuscular field also heavily relies on animal models like the *mdx* model for Duchenne Muscular Dystrophy (DMD) to better analyze the effect of treatment in a more physiological context. We included the study by Dort et al., that explores the potential of a Gpr18 agonist, PSB-KD107, to mimic the effects of Resolvin D2 on mouse myoblasts which promotes activation of the M2 macrophages. Gpr18 agonist activation of fatty acid metabolism and signaling pathways is crucial for resolving inflammation. Additionally, their findings indicate that Gpr18 agonists have the potential to stimulate myogenesis by targeting myogenic cells.

In recent years, some investigations have shown the significance of pursuing approaches that focus on underlying aspects of the disease which may not be their primary etiology. Indeed, identifying and tackling modulators of the disease could improve symptoms or affect its course. The studies by Navarro-Martinez et al., Ulm et al., highlights the need to expedite the repurposing of already available drugs. These studies show that using existing data from distinct disease models, but sharing common mechanisms, could potentially speed up the discovery of potential therapies.

The review by Navarro-Martinez et al. aims to highlight potential novel candidate genes that may be causative, or modifiers of neuromuscular junction-related disorders based on data from already-published mouse models.

Ulm et al. demonstrate that drug repurposing might be accelerated for DMD based on semantic linkages by comparing the earliest year when a drug was identified as a DMD treatment candidate with the first confirmed report of efficacy in the last few decades. Building up on this, they used Swanson-linking approaches and curated the DrugBank database to identify new promising prospects, including potential new ACEi/ARBs or Histone Deacetylase Inhibitors.

Clinical trials in rare diseases require careful planning and preparation to ensure success. Key considerations include selecting meaningful endpoints, considering the heterogeneity of these diseases, and ensuring sufficient powering for limited participants. Regulators may request commonly used outcome measures from more prevalent disorders, but differences in disease mechanisms can render these measures inappropriate leading to masking of clinical benefits.

Here we present the results of a Phase 2 clinical study by Mendell et al. for Delandistrogene moxeparvovec, an AAV gene therapy delivering a mini dystrophin gene for the treatment of DMD. The-study shows robust mini-dystrophin protein expression up to 60 weeks post-treatment, with a favorable benefit-risk profile and motor function stabilization sustained over 2 years in ambulatory patients aged 4 to 5 but not for older boys aged 6 to 7 in comparison to a control cohort. This highlights the necessity to ensure that the study design of clinical trials in rare disease is well balanced and optimized despite the limitations inherent to a small group of eligible patients.

In summary, the works collected here highlight challenges and approaches towards development of novel more personalized treatments for neuromuscular diseases. To advance the field, we need to tackle these rare diseases on multiple fronts. Therefore, it is imperative to collect more detailed neuromuscular disease natural history data, to develop relevant patient and mutation specific models and to explore alternative treatments beyond the obvious pathways. Once a candidate has been identified, the clinical trial design and selection of a patient population with most chance of therapeutic benefit are key elements to success. This Research Topic of articles discuss some key elements to understand the etiology and the raising of personalized medicine for rare neuromuscular diseases.

